# Enhancement of Reproductive Heat Tolerance in Plants

**DOI:** 10.1371/journal.pone.0122933

**Published:** 2015-04-07

**Authors:** John J. Burke, Junping Chen

**Affiliations:** USDA-ARS Cropping Systems Research Laboratory, 3810 4^th^ Street, Lubbock, Texas, United States of America; Texas Tech University, UNITED STATES

## Abstract

Comparison of average crop yields with reported record yields has shown that major crops exhibit annual average yields three- to seven-fold lower than record yields because of unfavorable environments. The current study investigated the enhancement of pollen heat tolerance through expressing an Arabidopsis thaliana heat shock protein 101 (*AtHSP101*) that is not normally expressed in pollen but reported to play a crucial role in vegetative thermotolerance. The *AtHSP101* construct under the control of the constitutive ocs/mas ‘superpromoter’ was transformed into cotton Coker 312 and tobacco SRI lines via Agrobacterium mediated transformation. Thermotolerance of pollen was evaluated by in vitro pollen germination studies. Comparing with those of wild type and transgenic null lines, pollen from *AtHSP101* transgenic tobacco and cotton lines exhibited significantly higher germination rate and much greater pollen tube elongation under elevated temperatures or after a heat exposure. In addition, significant increases in boll set and seed numbers were also observed in transgenic cotton lines exposed to elevated day and night temperatures in both greenhouse and field studies. The results of this study suggest that enhancing heat tolerance of reproductive tissues in plant holds promise in the development of crops with improved yield production and yield sustainability in unfavorable environments.

## Introduction

Comparing with reported record yields, the average yields of major crops grown in the U.S. represented only 22% of the mean record yield because of unfavorable environments [1]. Crops with economically valuable reproductive structures showed the greatest discrepancy between average and record yields. Evaluation of crop losses between 1948 and 1989 by the Federal Crop Insurance Corporation showed that on average, 69% of insurance indemnities could be attributed to drought and excess heat in barley, corn, forages, oat, peanut, rye, safflower, soybean, and wheat. These data suggest that plants have high productivity potentials, but are operating well below their genetic potential, mostly due to high sensitivity of the reproductive structures to heat and drought stresses.

Pollen has been reported to be sensitive to elevated temperatures [2–11], and is the only plant organ that has insufficient induction of heat shock proteins in response to heat stress [12–14]. Exposure to elevated temperatures during flower development has also been shown to inhibit pollen formation and induce flower sterility [15,16]. Heat sensitivity of pollen occurs not only throughout its’ development prior to dehiscence, but also following dehiscence [17–19].

In the “In This Issue” review entitled HSP101: A Key Component for the Acquisition of Thermotolerance in Plants, Gurley [20] stated that “the finding that constitutive expression of *HSP101* can nearly eliminate the need to precondition plants to survive severe heat stress has important implications for efforts to improve stress tolerance in plants”. He hypothesized “engineering the expression of *HSP101* in pollen might improve fertilization during periods of thermal stress, although additional HSPs may also need boosting because pollen does not show the normal HS response [13,21–23]”. The present study investigated the effects of overexpressing Arabidopsis *HSP101 (AtHSP101)* in tobacco and cotton pollen on heat tolerance of reproductive tissues and examined the potential benefit in yield component of these plants. Our results showed the enhanced pollen heat tolerance and improved yield production in *AtHSP101*transgenic plant lines.

## Material and Methods

### Construct and plant transformation

**Fig 1:**
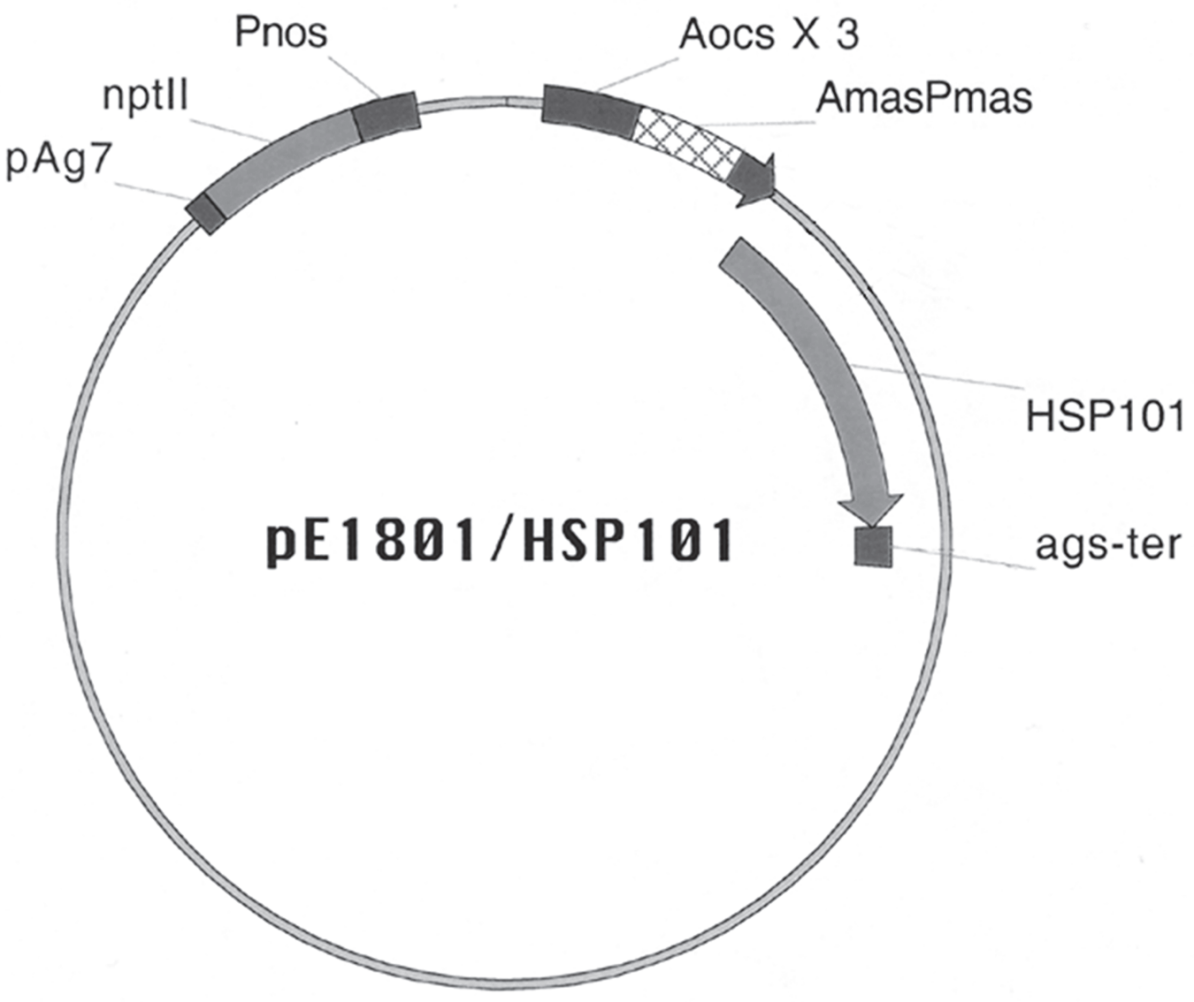
Diagram of pE1801-ocs/mas ‘superpromoter’-HSP101 plasmid. **PAg7** = Transcription termination and poly-Adenylation signal sequence from Octopine Ti-Plasmid T-DNA (gene for transcript #7) [57]; **NptII** = Neomycin phosphotransferase II coding region [58]; **Pnos** = Nopaline synthase promoter from Nopaline Ti-Plasmid T-DNA [59]; **Aocs X 3** = Octopine synthase enhancer element (3 copies) from Octopine T-Plasmid T-DNA [60]; **AmasPmas** = Manopine synthase promoter from Octopine Ti-Plasmid T-DNA [61]; **HSP101** = Heat shock protein (101 kdalton molecular weight) from *A*. *thaliana* [32]; **Ags-ter** = Transcription termination and poly-Adenylation signal sequence from Octopine Ti-Plasmid T-DNA (Agropine synthase gene) [62].

Total RNA was isolated from 10-day-old Arabidopsis seedlings using the RiboPure Reagent according to manufacturer’s instruction (Ambion Inc., Austin, TX) and used to synthesize cDNA using oligo-T primer and the ProSTAR Ultra HF RT-PCR System from Stratagene (La Jolla, CA). The coding sequence of Arabidopsis HSP101 was PCR amplified using gene specific primers and cloned into the pBSK plasmid. The plasmid clones containing the correct sequences of *AtHSP101* gene were used to make a FL-cDNA construct under the control of the constitutive SuperPromoter [24] (Aocs x3-AmasPmas::AtHSP101) in the binary vector pE1801 (a kind gift of Dr. Stanton Gelvin, Purdue University). The sense construct, pE1801-*HSP101*, was identified by PCR amplification of Aocs x3-AmasPmas::HSP101 5-prime junction using specific primer sets (primers P-AGS-> [CCAATACATTACACTAGC] vs HSP101-5' [CGGTAATGTTGTAAAATTGATAAC], hsp101-3 [TGACTCTTTTGGTAGACTATAATG] vs Ags-T [CATCCCAATCTGAATATCC], NPT2-> [CTTGCTCCTGCCGAGAAAGTATC] vs NPT2<- []) and subsequently confirmed by sequencing (Fig 1). The plasmid construct was transformed into *Agrobacerium tumefaciens* strain EHA105 strain [25] and the presence of pE1801-*HSP101* in transformed *A*. *tumefaciens* was examined by PCR amplification and verified by sequencing.

### Cotton and tobacco transformation

The Aocs x3-AmasPmas::atHSP101 was introduced into cotton and tobacco tissue segments by *Agrobacterium*-mediated tissue culture transformation. Cotton hypocotyl sections of Coker 312 (*Gossypium hirsutum* L., Coker 312) and leaf segments of *Tobucum tobucum* cv SR1 were co-cultivated with transformed *A*. *tumefaciens*. Regeneration of transformed plants for cotton and tobacco were achieved using the procedures described Bayley et al [26] and by Horsch et al. [27], respectively. For tobacco, the transformants were first selected on kanamycin (100 pg/mL) medium and then further screened for the production of nopaline according to Otten and Schilperoort [28].

For cotton, callus induced after co-culture of the plant tissue with *A*. *tumefaciens* is a mixture of transformed and non-transformed cells as no selection pressure is applied during the ensuing callus growth. The callus is subsequently moved to a liquid cell-suspension medium and grown with shaking to promote embryogenesis. Resulting cell clusters and pre-embryos were transferred to a solid medium, followed by a timed exposure to elevated temperatures in a 50°C incubator to select for heat-tolerant genetic transformants according to procedure described by Burke et al. [29].

The presence of the Aocs x3-AmasPmas::hsp101 5’ junction, hsp101::Ags-Terminator 3’ junction, and/or the NPTII selectable marker within transgenic embryos and/or plantlet generated was examined by PCR amplification using specific primer sets and the “Extract-N-Amp” plant PCR kit (Sigma-Aldrich, St Louis, MO). Possible contamination of transgenic plants by *A*. *tumefaciens* was examined by PCR amplification using primer sets targeting the non-transferred VirD region of the Ti plasmid (Genbank NC_003065). A primer set specific for the native cotton cellulose synthase gene (*ces1A*, GenBank:AY632360) was used as quality controls for PCR amplification using genomic DNA samples. Additional PCR analyses of transgenic plant lines were performed according to the procedure of Xin et al. [30].

The transgenic plantlets identified were transferred to Sunshine Mix #1 soil (Sun Gro Horticulture Distributors Inc., Bellevue, WA) and allowed to set T1 seeds in greenhouse under optimal conditions set at 31°C /27°C day/night temperatures and a 16/8 h photoperiod cycle. The supplemental lighting was provided by 430 W high-pressure sodium lights (P. L. Light Systems, Canada) to maintain a 16/8 h photoperiod. All plants were fertilized with Peters Excel fertilizer (Scotts-Sierra Horticultural Products Company, Marysville, OH) through the automated watering system.

The copy numbers of Aocs x3-AmasPmas::atHSP101 in each transgenic line were determined by the segregation ratio of T2 seedlings for the growth on selection medium and for PCR product of Aocs x3-AmasPmas-HSP101 5’-junction. Transgenic lines containing a single copy insertion were selected for further characterization.

### Semi-Quantitative RT-PCR and Western analysis

Total RNA was isolated from plant pollen or leaf samples collected using the RiboPure Reagent according to manufacturer’s instruction (Ambion Inc., Austin, TX) and used for semi-quantitative, RT-PCR amplification. Equal amounts of total RNA (500 ng) were used to synthesize cDNA using oligo-T primer and the ProSTAR Ultra HF RT-PCR System from Stratagene (La Jolla, CA). RT-PCR assays were performed using the cDNAs as templates and gene specific primers for AtHSP101 (AT1G74310, according to manufacturer’s instruction. PCR of the cotton actin gene was used for normalization between samples. Equal volumes of PCR reactions from plant samples were separated on 1.5% agarose gel and photographic exposure time adjusted to show the quantitative differences.

Total protein of individual plant samples was extracted in extraction buffer (100 mM NaPO4 pH 7.0 and 100 mM NaCl). The supernatant was then mixed with equal volume of 2X SDS sample buffer (Laemmli, 1970) and boiled for 10 minutes. Protein concentration was determined according to Bradford (Bradford, 1976) using bovine serum albumin as a standard. Fifty μg of total proteins were separated on 8% SDS/PAGE (W/V) and then blotted to PVDF membrane (Bio-Rad, Hercules, CA). Western blot was performed with anti-HSP101 antiserum at a dilution of 1:1,000 (v/v), which was visualized using an immune-blot kit (Bio-Rad, Hercules, CA)

### Hypocotyl elongation thermotolerance assay in tobacco

Hypocotyl elongation of wild type tobacco (SR1) and selected transgenic lines were examined according to procedures described by Hong and Vierling [31]. Sterilized tobacco seeds were plated in single seed rows on 1% Phytagar plates. The plates were then placed in a vertical position in an incubator set to 22°Cand incubated in dark for 7 days. The germinated seedlings were then treated at 50°C for 5 min followed by a 16 h growth at 22°C. The hypocotyl elongations of treated and non-treated seedlings were then characterized and photographed.

### Thermotolerance assay for in vitro pollen germination

All pollen germination experiments were performed according to the procedures described by Burke et al. [17] unless where is specified otherwise. For tobacco, flowers were collected from SR1 wild type and transgenic plants grown at optimal conditions, placed into pre-moistened containers and brought to the lab immediately. Upon arrival to the lab, pollen were dusted onto the germination plates [17] and allowed to germinated under one of the following 4 treatment conditions: 1) 30°C for 3 hours; 2) 46°C 3 hours; 30°C for 1 hour; and 4) 50°C for 7 minute followed by 30°C for 53 minutes. At the end of treatment, pollen germination and pollen tube growth were captured microscopically using an Olympus BX60 microscope (Olympus Optical Co., Japan) equipped with a MTI 3 CCD camera system (PAGE-MTI Inc, Michigan City, IN) and saved as tiff files. A minimum of five plates per treatment was evaluated and the experiments were repeated 3 times.

The thermotolerance assays for cotton pollen germination were performed under three different temperature treatment regimes. 1) Pollen was germinated at optimal temperature of 28°C after a five hours treatment of the harvested flowers at either 23°C or 37°C. 2) (2) A directly heat-challenged pollen from flowers of greenhouse (31°C/27°C, optimal condition) grown plants by germination for one hour at either 23°C (control) or 39°C (heat-treated). 3) Pollen from plants grown under either a 31°C/27°C day/night temperatures greenhouse or a 43/28°C day/night temperatures greenhouse was examined for germination at 28°C.

### Greenhouse study

Seeds from wild type Coker 312, AtHSP101 transgenic and null-transgenic lines were planted into 30 cm diameter pots containing Sunshine Mix #1 soil (Sun Gro Horticulture Distributors Inc., Bellevue, WA), 3 seeds per pot. Seedlings were thinned one week following planting to one plant per pot. For temperature treatment, five pots per line were placed on benches in greenhouses set to provide either 31/27°C or 43C/28°C day/night temperatures with a 16h/8h photoperiod. All other growth conditions such as light and nutrient levels were maintained the same for both greenhouses. The pots in each greenhouse were grouped as one pot per line and placed on different benches in the greenhouse. After entering reproductive stages, flowers were harvested at the day of opening for pollen germination study. Thirty-five days after first flowering, cotton bolls were harvested. Number of bolls per plant, number of seeds per plant and other yield related parameters were recorded.

### Field study: 2007

15-meter rows of AtHSP101 Plus Coker 312 and AtHSP101 minus Coker 312, were planted in an East-West orientation on Day of Year 123 (May 3, 2007) in field 103 at the University of Arizona, Agricultural Experiment Station in Maricopa, AZ under APHIS notification #07-082-104n. Standard cultural practices were utilized throughout the experiment. The plants were maintained in a well-watered state by furrow irrigation. Twenty individual plants were randomly selected and mapped for boll production on Day of Year 271 (September 28, 2007).

The daily maximum and minimum air temperatures for August and September 2007 in Maricopa, Arizona were obtained during the experiment from the monthly data located on Weather.com.

## Results

### Characterization of tobacco and cotton transformants

**Fig 2:**
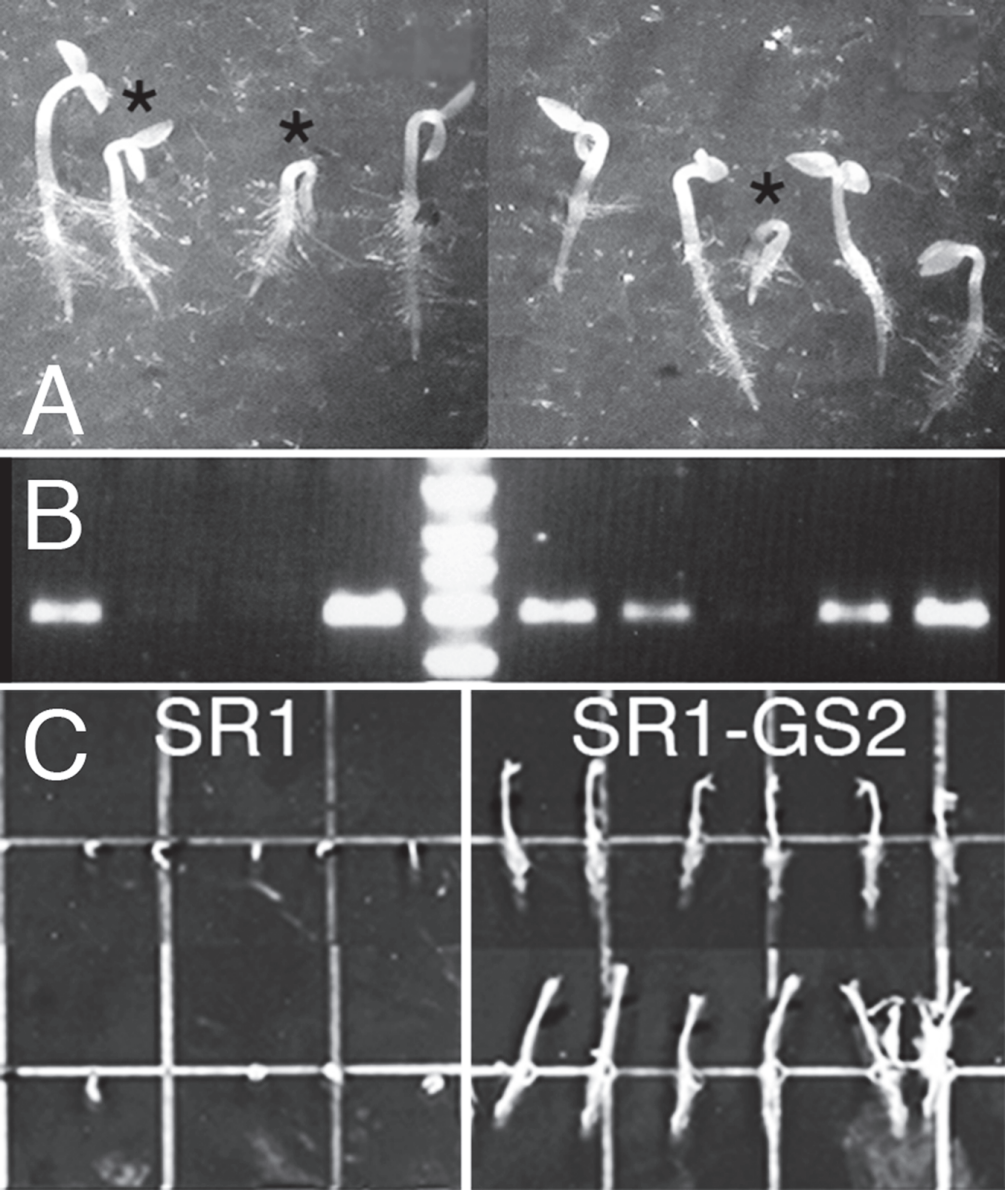
Tobacco seedling responses to a high temperature challenge. (A) Photograph of segregating SR1-GS24 F2 tobacco seedlings that had been heat treated for 5 min at 50°C and returned to the dark at 22°C for 16 hours. The seedlings with the asterisk above them were negative when analyzed for AtHSP101 by PCR. (B) PCR analysis of the segregating SR1-GS24 F2 tobacco seedlings shown in (A) using P-AGS-> HS101-5’<- primers. (C) Photograph of the characterization of tobacco heat tolerance using the hypocotyl elongation assay on SR1 (AtHSP101 minus) and SR1-GS2 (AtHSP101 plus) seedlings following a 5 min 50°C heat challenge and 16 h recovery. These assays were used to check for line homogeneity.

Initial studies of tobacco utilized the hypocotyl thermotolerance bioassay to evaluated *HSP101* expression and activity [31,32]. Fig 2A shows segregation of SR1–GS24 F2 seedlings for hypocotyl elongation phenotype after a five-minute heat treatment at 50°C followed by a 16-hour growth in the dark at 22°C. As expected, there was genetic diversity for heat shock response among the segregating seedlings. The seedlings that were sensitive to heat stress treatment showed reduced hypocotyl elongation (asterisk in Fig 2). PCR amplification of a segment of *AGS*::*AtHSP101* was performed to examine the presence or absence of the transgene in these seedlings (Fig 2B). Results showed a correlation between the presence of *AtHSP101* in transgenic lines and enhanced heat tolerance observed in hypocotyl elongation assay. Seedlings that exhibited improved heat tolerance contained the *AtHSP101* transgene whereas seedlings exhibiting heat sensitivity were null plants and did not contain the transgene (Fig 2A and 2B). Subsequently, the hypocotyl elongation assay was used to identify homogeneity within SR1-transgenic tobacco lines (Fig 2C).

**Fig 3:**
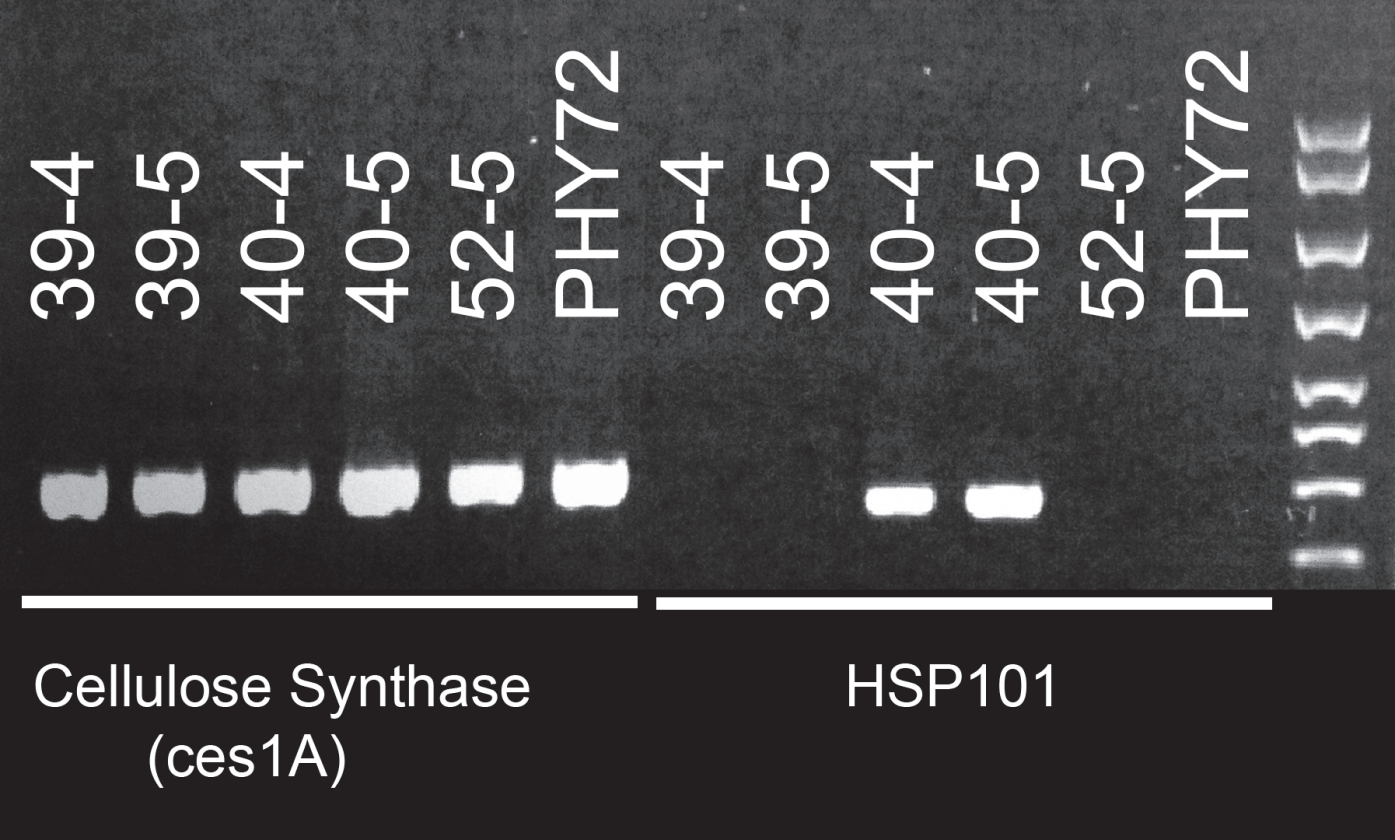
Genomic PCR analysis of cellulose synthase and AtHSP101 in segregating transgenic cotton lines and a commercial check (Phytogen 72).

PCR amplification of *AGS*::*AtHSP101* segment was used to characterize putative transgenic cotton lines initially. A representative PCR analysis of cellulose synthase and *AtHSP101* in segregating transgenic cotton lines and a commercial check (Phytogen 72) is shown in Fig 3. PCR amplification of cellulose synthase gene was included as a positive control for genomic DNA quality. As indicated, F2 plants of null lines 39, 52 and the Phytogen 72 check did not contain the *AtHSP101*, while transgenic line 40 exhibited the *HSP101*. All lines used in this study were evaluated for homogeneity.

### Expression of HSP101 in pollen

**Fig 4:**
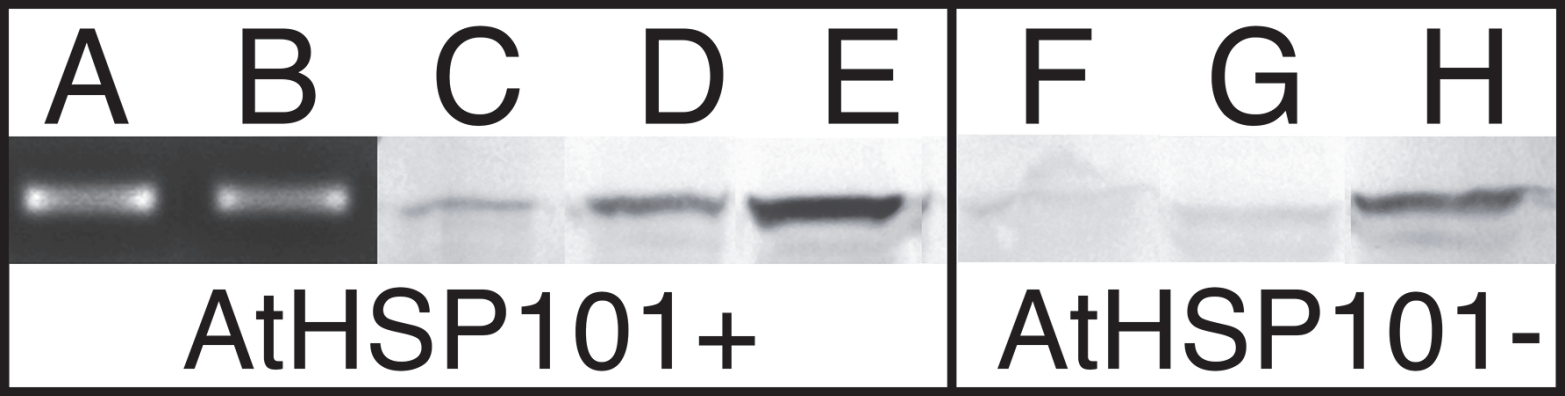
Photographs of RT-PCR and Western Blot analysis showing the expression of *AtHSP101* and presence of HSP101 proteins in transgenic cotton lines. RT-PCR products from pollen (A) and leaf (B) tissues of transgenic line 40 plants grown in 31°C/27°C greenhouse; Western blot analysis of HSP101 for: mature pollen of transgenic line 40 (C) and null line 52 (F, AtHSP101 minus) plants grown in a greenhouse set to 31°C/27°C day/night; leaf tissues of transgenic line 40 (D) and null line 52 (G) plants grown in a greenhouse set to 31C°/27°C day/night; leaf tissues of transgenic line 40 (E) and null line 52 (H) plants grown in a hot greenhouse set to 43°C/28°C day/night temperatures.

RT-PCR was performed to determine if the *AtHSP101* was expressed in mature cotton pollen of transgenic lines. The results showed accumulation of *AtHSP101* transcript in both pollen and leaf tissues of transgenic plants (Fig 4A and 4B) under normal growth temperatures, indicating the constitutive expressing nature of the transgene in these tissues. RT-PCR of Coker 312 and null line pollen came out negative (data not shown). Furthermore, we also examined AtHSP101 protein levels in transgenic cotton using Western blot analysis. Consistent with RT-PCR data, the protein results showed the accumulation of certain amounts of HSP101 in pollen (Fig 4C) and leaf (Fig 4D) tissues of transgenic line 40 plants grown under normal temperature conditions (31C/27C, d/n); whereas no noticeable amount of HSP101 proteins were detected in both tissue types of null line 52 plants (Fig 4F and 4G) grown under the same conditions. However, for plants grown in a hot greenhouse (43°C/28°C d/n), induction of HSP101 accumulations were observed in leave tissues of both transgenic and null transgenic lines (Fig 4E and 4H), with transgenic tissues accumulated in a much higher amount. The HSP101 band detected in null plants are likely to be cotton HSP101 homolog that was induced under the hot greenhouse conditions. The supportive evidence for such assumption came from our previously study where induction of maize HSP101 homolog by high temperatures was analyzed by the same HSP101 antibody (Chen et al, 2010). The high levels of HSP101 detected in leaves of transgenic cotton under hot conditions (Fig 4E) would be the combined amounts of transgene product of *AtHSP101* and cotton HSP101 homolog induced under hot conditions. Similar results were obtained in the characterization of tobacco transgenic plant lines.

### Enhanced pollen thermotolerance

**Fig 5:**
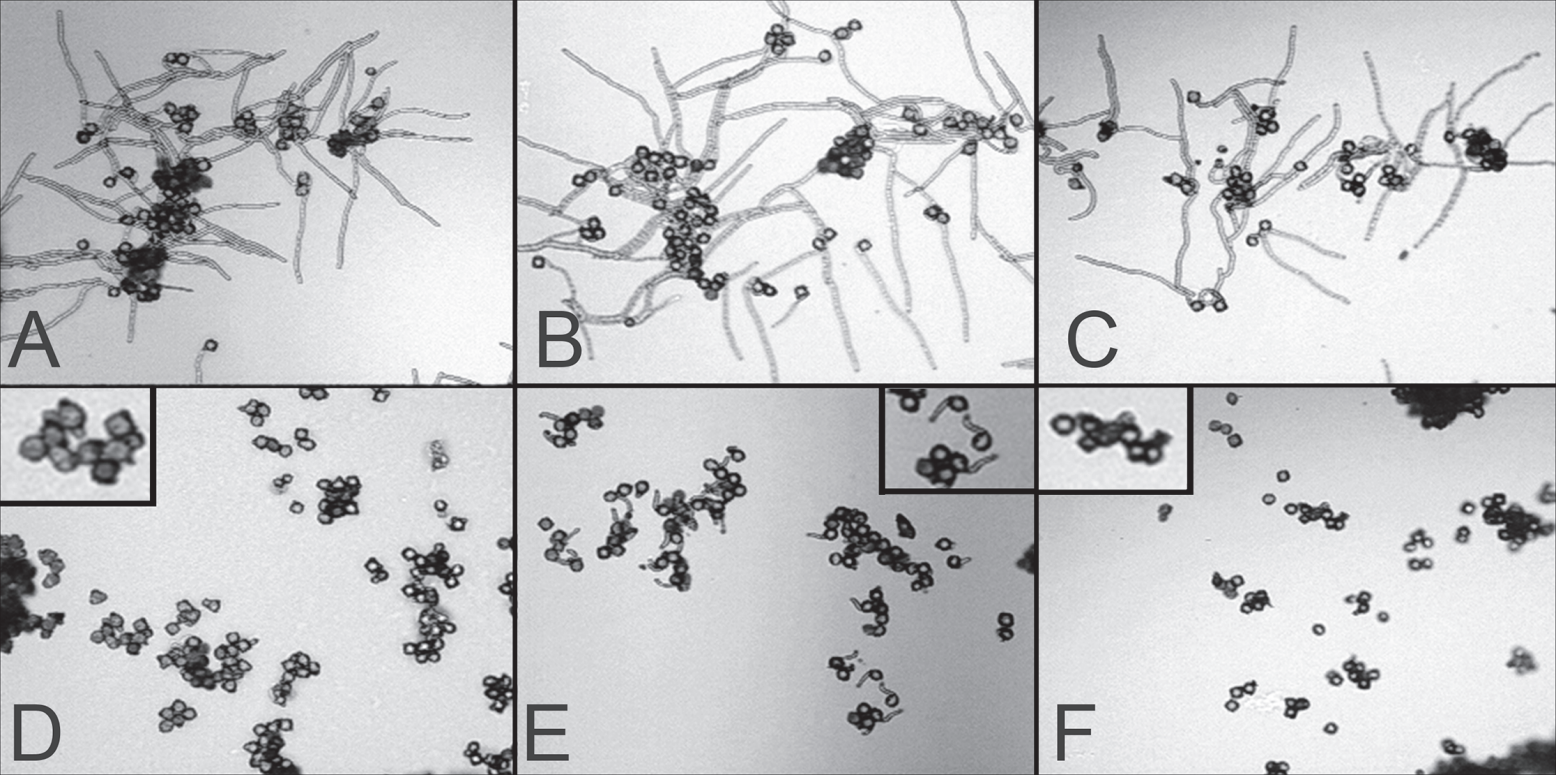
Photomicrographs of tobacco pollen following a high temperature challenge. SR1 (A), SR1-GS2 (B), and SR1-GS3 (C) tobacco pollen germination when incubated at 30°C for 3 hours; and SR1 (D), SR1-GS2 (E), SR1-GS3 (F) pollen germination when incubated at 46°C for 3 hours.

**Fig 6:**
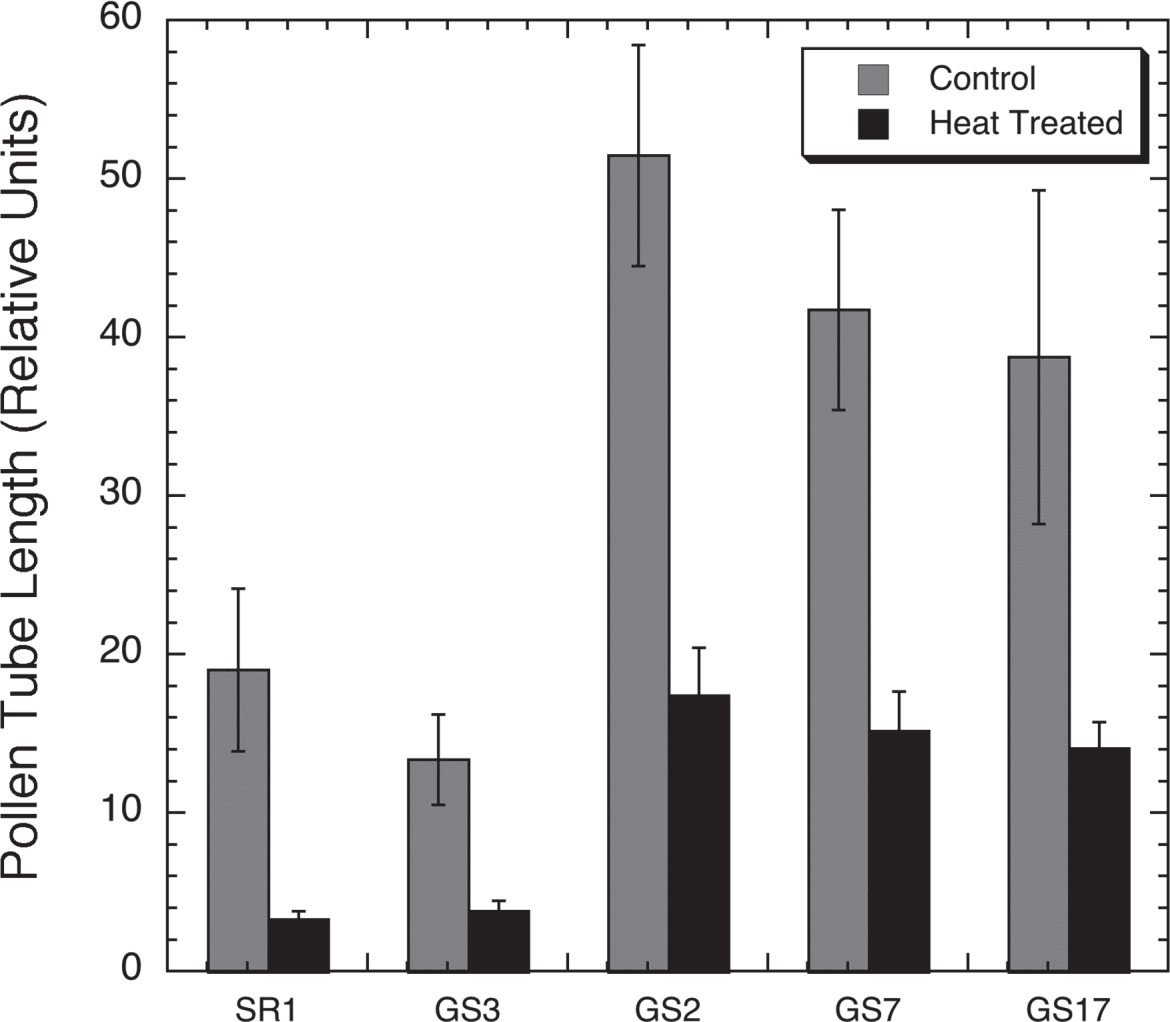
Tobacco pollen germination following a high temperature challenge. Graph comparing the relative pollen tube lengths of SR1, SR1-GS3, SR1-GS2, SR1-GS7, and SR1-GS17 tobacco pollen following a 60-min incubation at 30°C (control—white bars) or 7 min at 50°C followed by 53 min at 30°C (heat treated—black bars).

To determine if expression *AtHSP101* in pollen affects pollen viability and growth, we examined pollen germination rate and pollen tube elongation of transgenic tobacco GS2, GS7 and GS17 lines and compared the results with those of wild-type SR1 and a null GS3 line. No difference in pollen germination rate was observed among SR1 (Fig 5A), transgenic (Fig 5B) and null (Fig 5C) lines under optimal temperature condition. Nevertheless, noticeable variations in the degree of pollen tube elongation were observed among these lines (Figs 5A–5C and 6). Comparing with SR1 WT, the pollen tube elongation in GS3 null line was slightly reduced (Figs. 5C and 6). Conversely, transgenic lines such as GS2, GS7, and GS17 had much vigorous pollen tube growth under control conditions with a doubling of tube lengths compared to the SR1 control (Figs. 5B and 6). The enhancement of pollen tube growth was observed in all transgenic lines examined under normal conditions.

The possible enhancement of thermotolerance of pollen in AtHSP101 transgenic tobacco plants was examined in two different pollen germination assays. First, pollen germination was examined after a 3-hour incubation at either optimal (30C) or high (46C) temperature on germination medium. Fig 5 shows the observed pollen germination phenotypes of wild type, transgenic and null line pollen under both temperature treatments. As expected, all lines evaluated exhibited excellent pollen germination and pollen tube elongation when incubated at 30°C for three hours (Fig 5A–5C). The 3-h, 46°C heat treatment severely inhibited pollen germination and pollen tube elongation (Fig 5D–5F) in all lines examined. However, after 3-hours exposure to 46°C, pollen of transgenic GS2 line exhibited noticeable pollen germination and pollen tube elongation (Fig 5E) while pollen germination and pollen tube growth were not evident for both wild-type and null plants (Fig. 5D and 5F), suggesting enhanced heat tolerance in pollen of *AtHSP101* transgenic plants.

**Fig 7:**
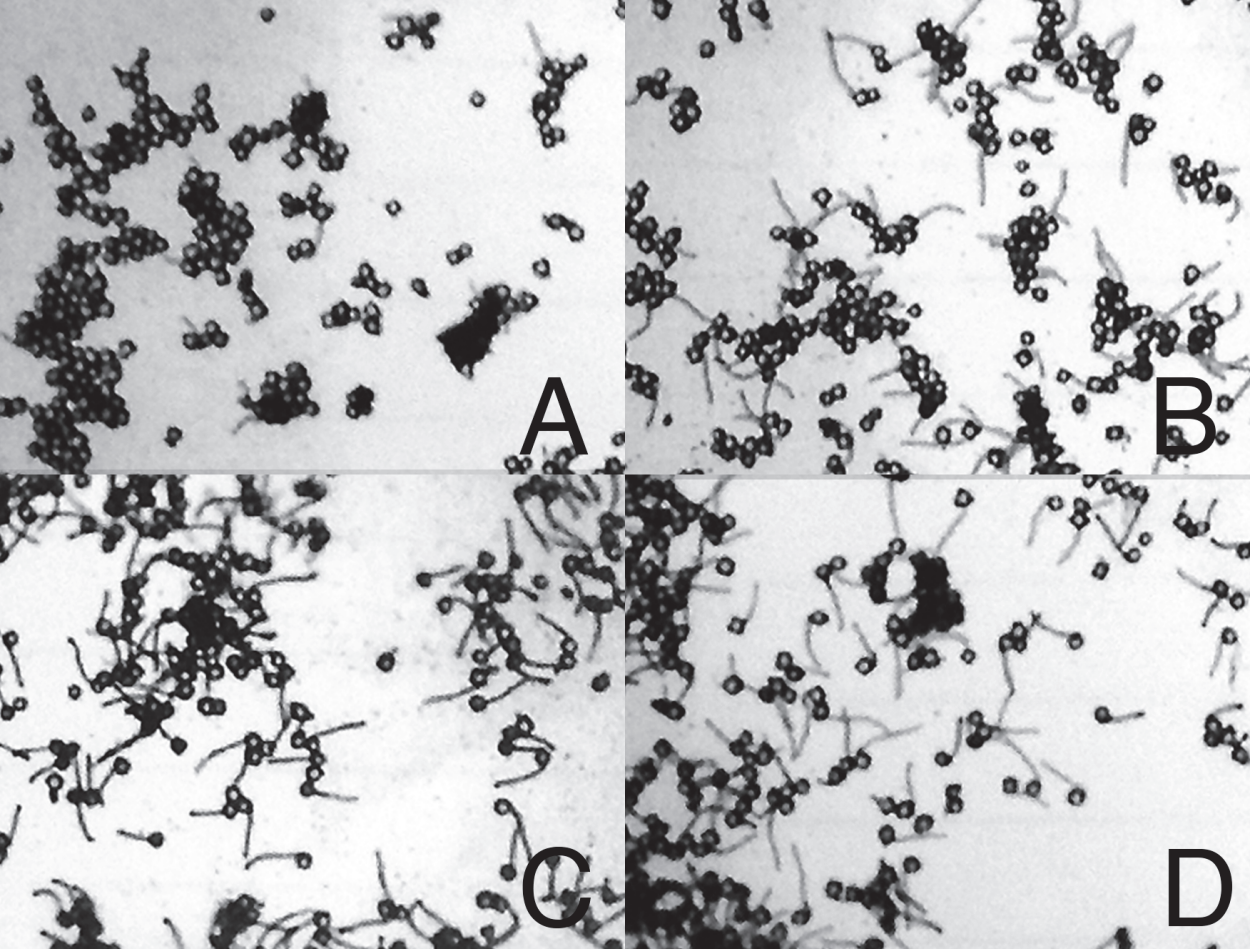
Photomicrographs of tobacco pollen following a high temperature challenge. SR1 (A), SR1-GS2 (B), SR1-GS7 (C), and SR1-GS17 (D) tobacco pollen incubated for 7 minutes at 50°C and then 53 minutes at 30°C.

The second assay performed for pollen heat tolerance was to treat tobacco pollen to a 7-min, 50°C heat exposure followed by a 53-min incubation at normal temperature of 30°C. Pollen of transgenic lines and non-transgenic controls (wild-type and null) exhibited striking differences in pollen germination after the heat treatment (Figs 6 and 7). Pollen germination rates of transgenic GS2, SGS7, and GS17 were significantly higher those of control lines. The brief 7-min heat exposure also caused significant reductions in pollen tube growth in all lines examine (Fig 6). However, the degree of such reduction for pollen tube elongation differed significantly between transgenic lines and the control lines (Fig 6). While pollen tube growth in wild type SR1 was very much inhibited by such a brief heat treatment (Fig 7A), pollens of *AtHSP101*transgenic lines showed a much greater pollen tube elongation under the same treatment (Fig 7B–7D). Quantitatively, pollen tube lengths of heat-treated SR1 and GS3 pollen were only 16% and 23% of those non-heat treated pollen respectively. For transgenic lines, the average pollen tube lengths of heat-treated pollen were 36 to 38% of those non-treated pollen. Moreover, despite the reduction in pollen tube growth caused by heat treatment, pollen tube lengths of heat-treated transgenic GS2, GS7, and GS17 pollens were comparable with those of SR1 and GS3 pollen germinated under optimal conditions (Fig 6).

**Fig 8:**
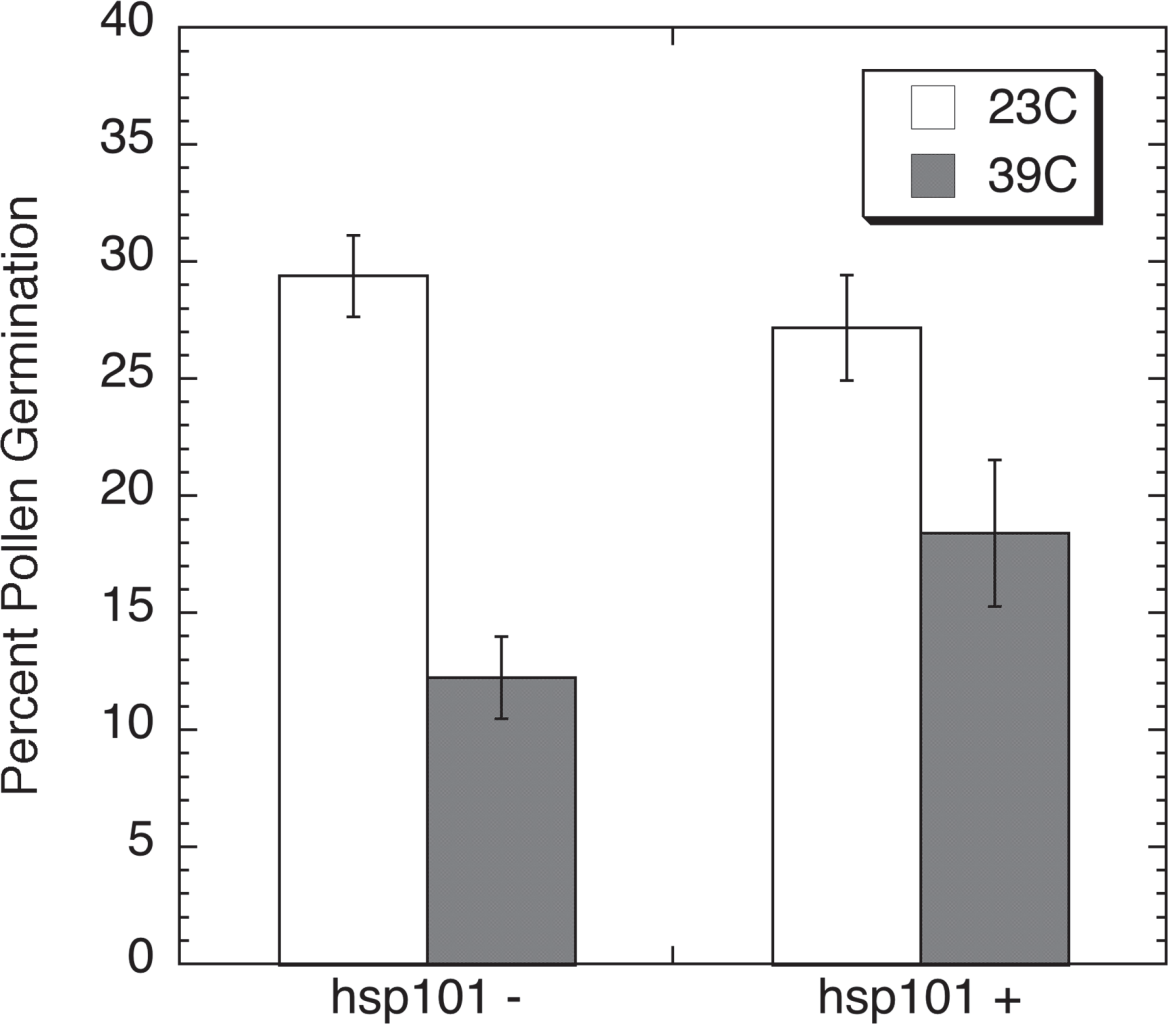
Cotton pollen germination following a high temperature challenge. Graph showing the percent germination of control (AtHSP101 minus, plant 39) and transgenic (AtHSP101 plus, plant 40) pollen from greenhouse grown cotton germinated for one hour at either 23°C or 39°C on the solid media described by Burke et al. [17]. Error bars represent Standard Deviation and the sample size was n = 20, p value is 0.048388.

**Fig 9:**
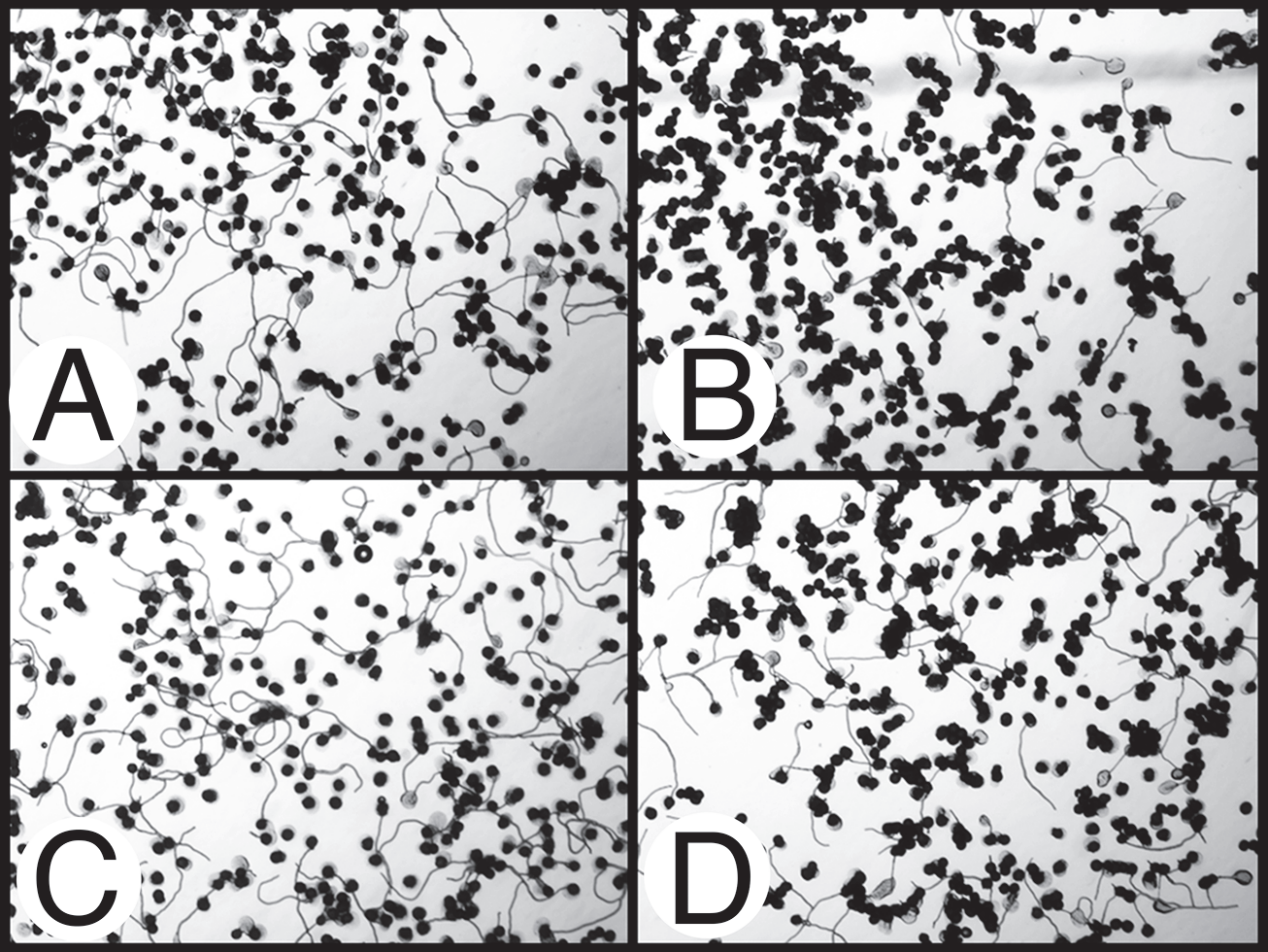
Photomicrographs of cotton pollen following a high temperature challenge. (A) pollen from plant 39 (AtHSP101 minus) germination at 28°C after treating flowers for five hours at 23°C, (B) pollen from plant 39 (AtHSP101 minus) germination at 28°C after treating flowers for five hours at 37°C, (C) pollen from plant 40 (AtHSP101 plus) germination at 28°C after treating flowers for five hours at 23°C, and (D) pollen from plant 40 (AtHSP101 plus) germination at 28°C after treating flowers for five hours at 37°C.

The positive impacts of expressing *AtHSP101* on heat tolerance of cotton pollen were also observed in transgenic cotton lines in 3 different heat stress treatments. First, pollen from 31/27°C grown cotton were germinated for one hour at either 23°C or 39°C and the germination rates among transgenic and null-transgenic control lines were examined and compared. The result showed no significant differences in pollen germination rates between *AtHSP101* transgenic and null lines under 23°C condition (Fig 8). However, at elevated temperature of 39°C, the percent of transgenic pollen germinated was significantly (p-0.048) higher than those of null line (Fig 8), a result similar to that of transgenic tobacco. The second heat tolerance assay performed was to incubate the cotton flowers collected from 31/27°C grown cotton plants for five hours at either 23°C or 37°C. Pollen of those treated flowers were collected at the end of 5-hr treatment and allowed to germinate for one hour at 28°C on germination medium. As shown in Fig 9, pollen from flowers of transgenic and null control lines exhibited similar levels of pollen germination and tube development after a 5-hr incubation at 23°C (Fig 9A–9C). However, clear differences in pollen germination were observed for pollen of heat-treatment flowers. Pollen of the heat-treated null control line (Fig 9B) exhibited much greater reductions in pollen germination and pollen tube growth than those of *AtHSP101* transgenic line (Fig 9D).

**Fig 10:**
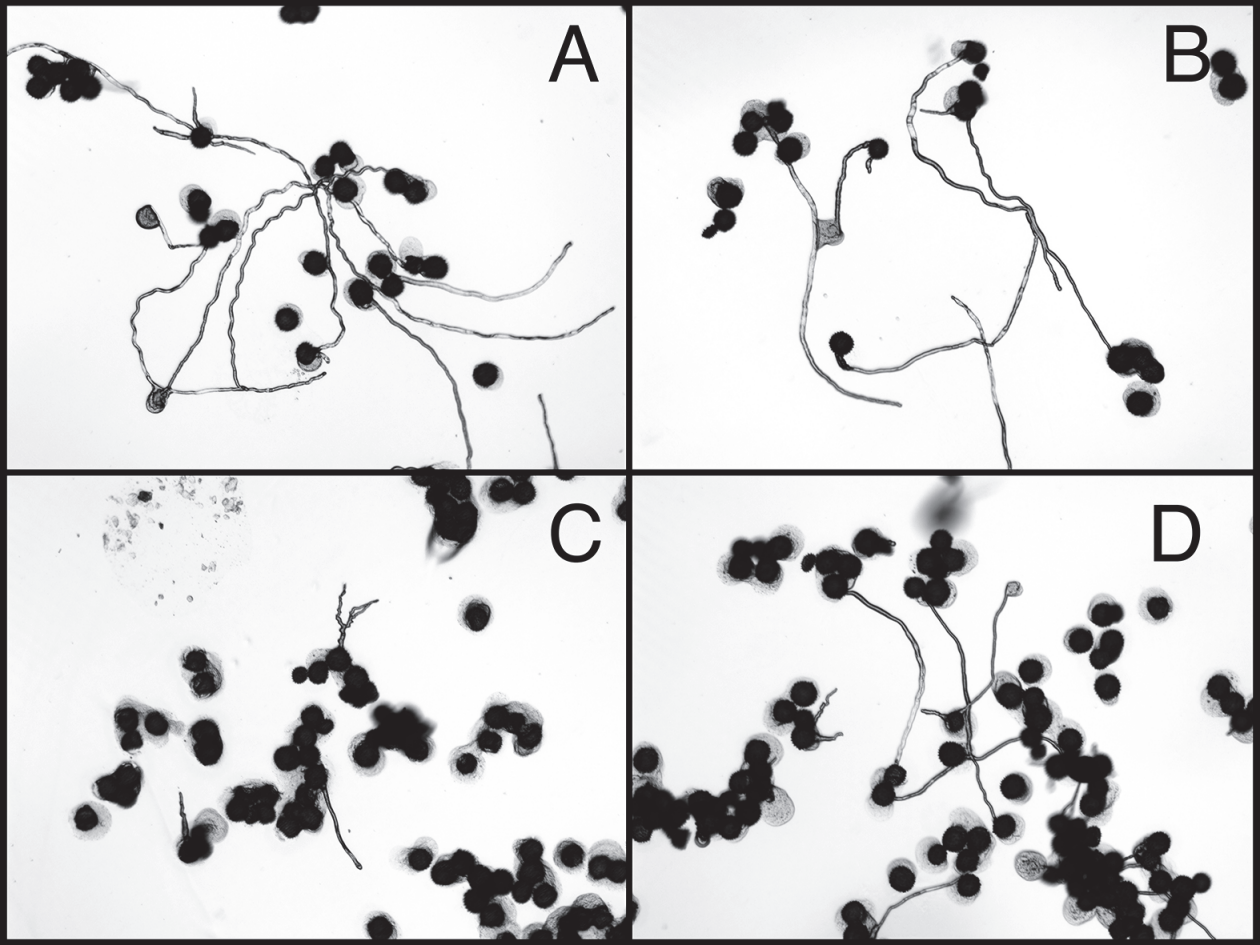
Photomicrographs of cotton pollen following a high temperature challenge. Representative photomicrographs showing pollen germination at 28°C according to the procedure described by Burke et al. [17] from plants grown under control temperatures (31°C/27°C day/night temperatures) and elevated temperatures (43°C/28°C day/night temperatures). (A) Plant 52 Control, (B) Plant 40 Control, (C) Plant 52 from a greenhouse with elevated day/night temperatures, (D) Plant 40 from a greenhouse with elevated day/night temperatures.

The observed differences in heat tolerance of isolated pollen in in vitro studies led us to evaluate pollen germination and pollen growth in vivo from plants grown under either normal temperature (31°C/27°C day/night) or elevated temperature (43°C/28°C day/night) conditions. Pollen collected from cotton flowers under both temperature conditions were all allowed to germinate for 1 hour at 28°C. Photographs of representative results were shown in Fig 10, similar pollen germination and pollen tube development were observed for pollen of both transgenic and null plants grown in the normal temperature settings (Fig 10A and 10B). Reductions in pollen germination and pollen tube growth were observed for pollen from plants grown under elevated temperature settings (Fig 10C and 10D). However, pollen from AtHSP101 transgenic lines showed significantly higher germination rate and greater pollen tube growth (Fig 10D) than those of null plants (Fig 10C). The observed enhancement for pollen heat tolerance in vivo in *AtHSP101* transgenic cottons was consistent with those of in vivo pollen germination studies in both transgenic tobacco and cotton lines (Figs 6–9).

### Enhanced boll production in elevated temperature conditions in greenhouse studies

The effect of expressing *AtHSP101* on cotton reproductive heat tolerance was evaluated by determining boll production on plants grown in either a greenhouse with control (31°C /27°C day/night) or heat-treated (43°C/28°C day/night) environments. Table 1 provides our findings on the bolls per plant, seeds per plant, and seeds per boll for homozygous transgenic plant lines of several independent transformants. No significant differences were observed in all yield components examined between AtHSP101 transgenic and null line plants grown in the control environment. Under elevated temperature conditions, however, AtHSP101 transgenic lines produced an average of 36 bolls per plant, while the AtHSP101 negative null lines produced 12 bolls per plant. Congruently, the numbers of seeds per plant produced by transgenic plants were significant higher than those of null plants in elevated temperature environment. The difference in seed production per plant was the results of relatively high boll retention in transgenic plants and was not related to the number of seeds per boll as shown in Table 1. The results indicate that cotton plants expressing the *AtHSP101* transgenes can increase yield gains under heat stress conditions. In addition, significant differences in the number of boll per plant and seed per boll were observed for plants grown under control and heat-treated environments. The heat-treated plants produced 9 to 10 seeds per boll, while the control plants produced 25 to 26 seeds per boll.

**Table 1:** Characterization of the heat tolerance of AtHSP101 plus and AtHSP101 minus cotton plants from different cell lines.

**Treatment**	Cell Line	Transgenic Plant Number	Bolls/Plant	Seeds/Plant	Seeds/Boll
**43°C/28°C day/night**	1–4 (pos.)	1	50	482	9.64
	1–4 (pos.)	2	51	418	8.20
	1–8 (pos.)	3	27	286	10.59
	1–8 (pos.)	22	29	251	8.66
	1–8 (pos.)	24	43	283	6.58
	21–2 (pos.)	5	18	142	7.89
	**Average**		**36**	**310**	8.59
	21–2 (neg.)	4	17	60	3.53
	C312-5A (neg.)	8	13	191	14.69
	6–9 (neg.)	10	10	110	11.00
	C312-5A (neg.)	18	23	220	9.57
	**Average**		**12**	**240**	9.70
**31°C /27°C day/night**	1–4 (pos.)	51	12	240	20
	1–4 (pos.)	50	18	424	23.56
	1–4 (pos.)	48	15	362	24.13
	1–8 (pos.)	40	22	631	28.68
	21–2 (pos.)	34	18	513	28.5
	21–2 (pos.)	35	31	703	22.68
	**Average**		19	479	25.2
	1–8 (neg.)	39	24	710	29.58
	1–8 (neg.)	52	14	352	25.14
	2–6 (neg.)	31	32	756	23.63
	**Average**		23	606	26.35

Values in boldface within a treatment are significant at a = 0.05 comparing AtHSP101 positive and AtHSP101 negative plants. Values underlined are significant at a = 0.05 comparing AtHSP101 positive and AtHSP101 negative plants between treatments.

Plants were grown in either a greenhouse with control (31°C /27°C day/night) or heat-treated (43°C/28°C day/night) environments.

**Fig 11:**
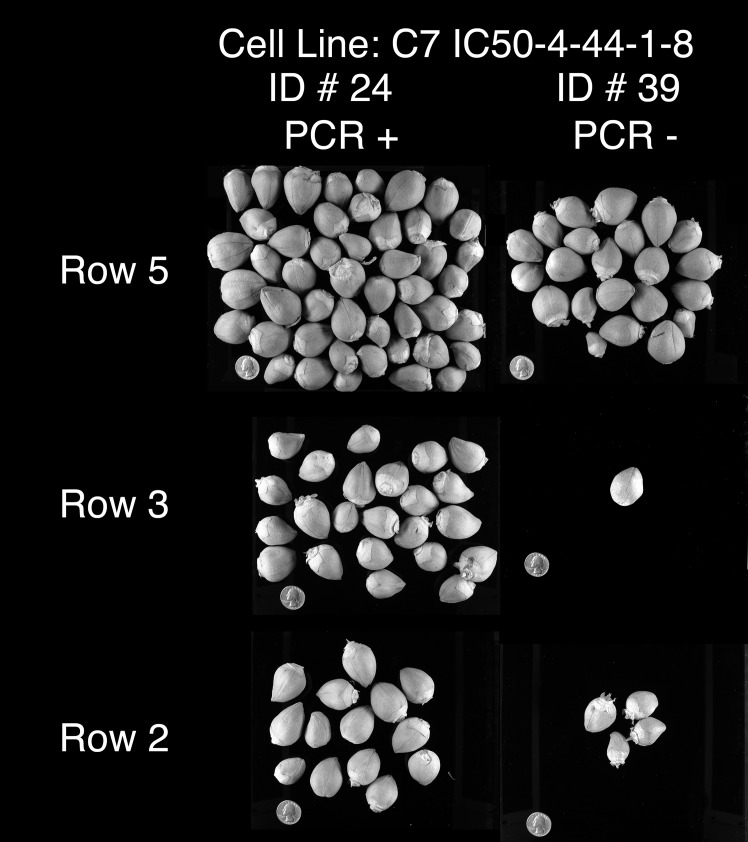
Boll accumulation on control and heat-treated cotton plants. Photograph of cotton bolls harvested from individual plants of plant line 24 (AtHSP101 plus) and plant line 39 (AtHSP101 minus) that were grown in a greenhouse with a 43°C/28°C day/night temperature regime. Paired plants (line 24 and 39) were randomly distributed throughout the greenhouse because of a measured uneven temperature distribution (set point ± 3°C) during the winter months.

**Fig 12:**
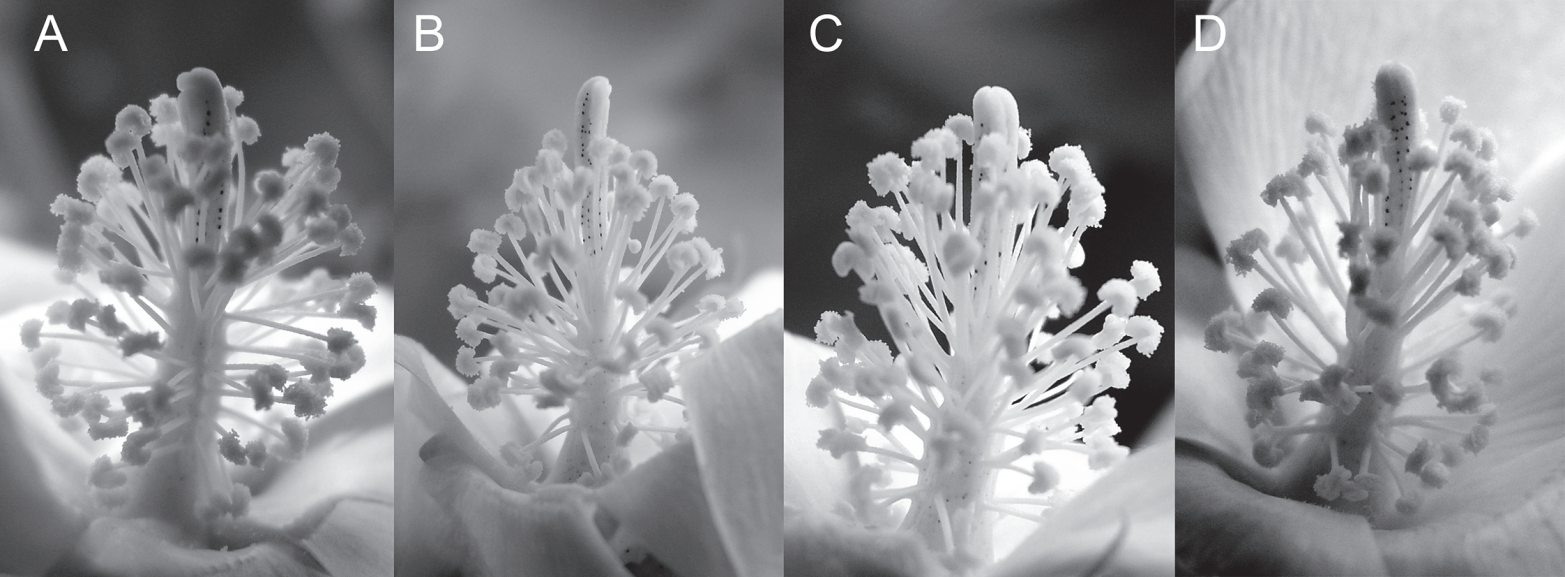
Photographs of anthers from high temperature grown cotton plants. (A) Line 39 [AtHSP101 minus], (B) Line 40 [AtHSP101 plus], (C) Line 52 [AtHSP101 minus], (D) Line 24 [AtHSP101 plus] flowers from plants grown under high temperatures (43°C/28°C day/night) in a greenhouse. Pollen dehiscence is apparent in all lines evaluated.

Plant lines from the transgenic line 1–8, described in Table 1, were used for further greenhouse studies on the effects of transgene on cotton production in heat-stressed environment. A summary of yield performance data for transgenic and null plants lines grown in a 43°C/28°C day/night temperature greenhouse environment is provided in Table 2. Null plant lines 39 and 52 yielded the fewest number of bolls and had the lowest fresh weight per boll. Transgenic plant line 24 produced the highest number of bolls with 30 bolls per plant. Transgenic plant line 40 had the highest fresh weight per boll, 6.35 gm versus 0.55 gm in null 39 plants. Based on these findings, null lines 39 and 52 and transgenic lines 24 and 40 were used for subsequent greenhouse studies. Paired plants of a transgenic and a null lines (24 and 39; 40 and 52) were randomly distributed throughout the greenhouse set for a 43°C/28°C day/night temperature cycle during the winter months. Two heating units located on opposite corners maintained the air temperature within the greenhouse. The measured uneven temperature distribution was set point plus/minus 3°C with hot spots close to the heating source. Fig 11 is a photograph of cotton bolls harvested from individually paired plants of transgenic 24 and null 39 lines. The impact of temperature variability within the greenhouse locations is apparent from the variability in boll set among replicate plants within a line. Plants near the heating source and directly in the path of hot air blown had much less boll set than those farther away, located indirectly from the heat source plants. Nevertheless, the positive impact of *AtHSP101* transgene on cotton boll production under heat stressed condition is very evident. In all paired cases/locations, plants of transgenic line 24 set more bolls than the adjacent plants of the null line 39. Similar result was observed in paired plants of transgenic 40 and null 52 lines.To determine if the observed difference in boll setting was partially due to high temperature induced flower sterility in the 43°C/28°C day/night environments, cotton flowers of transgenic and null plants were examined for pollen dehiscence. As shown in Fig 12, pollen dehiscence is apparent in all lines evaluated under hot (43°C/28°C) greenhouse conditions. The results suggest that the observed reductions in boll setting in hot greenhouse are mostly caused by heat sensitivity of mature pollen (Figs 8–10), and expression of *HSP101* in pollen can greatly enhance boll production of cotton plants under heat-stressed conditions (Fig 11).

**Table 2:** Characterization of the relative heat tolerance of AtHSP101 plus and AtHSP101 minus cotton plants from the 1–8 cell line.

**Transgenic Line**	Plant Number	HSP101	Bolls/Plant	Boll Fresh Weight/ Plant (gm)	Fresh Weight/Boll (gm)
1–8	39	-	11	6.1	0.55
1–8	40	+	17	108	6.35
1–8	52	-	13	14.2	1.09
1–8	3	+	17	42.7	2.51
1–8	24	+	30	86.5	2.88
1–8	22	+	13	29.8	2.29

Plants were grown in a greenhouse with a 43°C/28°C day/night environment.

### Enhanced boll production in transgenic cotton lines in a field study

**Fig 13:**
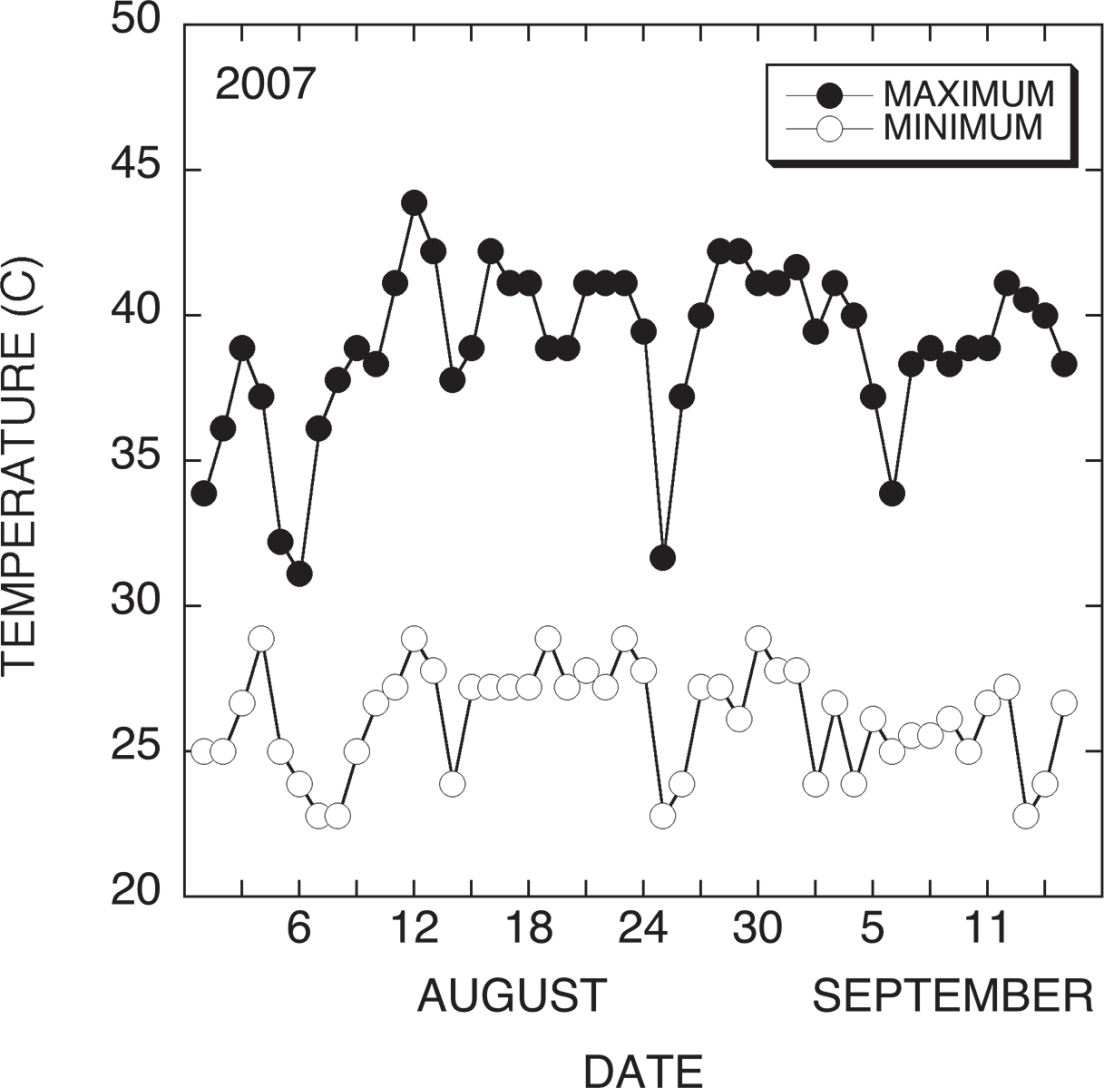
Field air temperatures during evaluation of boll accumulation. This graph shows the maximum and minimum air temperatures in Maricopa, Arizona during the 2007-growing season. Temperatures over 38°C were common throughout the flowering and boll development period. The Bar line indicates the time period where the opened bolls were set.

The apparent increase in heat tolerance of reproductive tissues in *AtHSP101* transgenic cotton lines was further evaluated under field heat-stress conditions in the field in Maricopa. Fig 13 shows the maximum and minimum air temperatures in Maricopa, Arizona during the peak of boll production in the growing season. Temperatures over 38°C were common throughout the flowering and boll development period. Table 3 provides the average data on the total number of bolls per plant, open bolls per plant, seed numbers per open boll, and closed bolls per plant for twenty plants for each of null line 39 and transgenic line 40 at the time of harvesting on September 27. The transgenic line 40 produced an average of 35% more bolls than the null 39 line. No significant difference in the number of open bolls or seeds per open boll was observed. The difference in total boll numbers was attributed to an increase in the number of closed bolls per plant. Overall, field results further demonstrated the enhancement of reproductive tissue heat tolerances in HSP101 transgenic cotton plants.

**Table 3:** Field performance of AtHSP101 minus (seed from plant #39) and AtHSP101 plus (seed from plant #40) cotton in Maricopa, Arizona during the 2007 growing season.

Seed Source	Bolls/Plant	Open Bolls/ Plant	Seeds/Open Boll	Closed Bolls/Plant
Plant 39 (AtHSP101 minus)	22.4	11.75	18.09	10.65
Plant 40 (AtHSP101 plus)	30.35	12.95	18.54	17.4
P value	0.08587	0.56197	0.561676	0.05484

Twenty plants per line were analyzed for total number of bolls per plant, open bolls per plant, seeds per open boll, and closed bolls per plant. Statistical analyses used an Unpaired Student's t test and the sample size was n = 20.

## Discussion

The report by Boyer [1] that crops with economically valuable reproductive structures showed the greatest discrepancy between average and record yields provided the impetus for evaluating ways to enhance reproductive stress tolerance. Mature pollen viability has been reported to be very sensitive to elevated temperatures [7,22,33–36] and this loss of viability has been associated with lacks of induction for heat shock proteins [13,21,37].

Young et al. [38] has reported that as maize pollen matures it progressively loses the ability to induce HSP101 production in response to heat stress. The study also found that the already low level of HSP101 protein in mature pollen appeared to decrease further following a heat stress. The study suggests that, with respect to HSP101, pollen may be only slightly heat responsive during its early development and this response is lost upon its maturation. Similarly, study of heat shock proteins in tobacco pollen also shows the lack or insufficiency inductions of HSP in pollen in response to a 2 h, 42°C treatment [14]. Proteomic analysis of tomato pollen indicate the presence of heat stress-related proteins at the very early stages of tomato pollen development and insufficiency of this class proteins in polarized microspore and mature pollens [39].

It is well documented that HSPs play critical roles in both acquired and basal thermotolerance in plants [20]. Heat shock protein is a class of molecular chaperones, important in maintaining cellular homeostasis by preventing the aggregation of denatured proteins, repairing misfolded proteins, and depredating unstable proteins damaged under heat stress conditions [40–42]. In seed, the presence of HSP101 has been related to basal thermotolerance during the first days following germination [20,32]. Mature pollen grains contain storage lipids and proteins similar to seeds but lack of HSPs. The deficiency in the amount of HSP in mature pollen and insufficiency of HSP induction in pollen upon heat stress may contribute to the high sensitivity of male reproductive tissues to high temperature stress. Hence, increase in the amount of HSP in pollen grains may enhance thermotolerance of mature pollen. The present study investigated the potential benefit of expression of *AtHSP101* in tobacco and cotton in the enhancement of thermotolerance in reproductive tissues of these plants.

Consistent with previous studies [14,43,44], our results show significant reductions in tobacco pollen germination and pollen tube growth under high temperature conditions. Kandasamy and Kristen have reported that tobacco pollen tube growth was decreased about 50% at 35°C and no growth of pollen tubes at temperatures of 40°C and above [43]. Authors postulated that the decrease of pollen tube growth with increasing temperature probably reflected the heat shock-induced inhibition of Golgi vesicles. Ultrastructural analysis indicated structural changes in the rough ER, Golgi apparatus and the mitochondria in response a sudden increase in growth temperature. The heat shock induced such changes persist in the pollen tubes grown at 35°C for about 24 h. They concluded that this indicated the heat shock effects once established, can persistent at least for 24 h, excluding the possibility of adaptation to the elevated temperature within the range of that period. We hypothesize that these structural changes induced heat shock may partially due to the insufficient amount of molecular chaperones HSP in mature pollens.

In this study, we have taken a transgenic approach and examined the effects of expression of *AtHSP101* in tobacco and cotton plants on pollen germination and pollen tube growth under evaluated temperatures in both in vitro and in vivo assays. Our study of tobacco transgenic plant lines have showed that pollen from the AtHSP101 containing transformants exhibit high germination percentages and excellent pollen tube development at 23°C (Fig 5 A–5 C), indicating normal growth and development of pollen in those transgenic lines. Consistent with previous reports [14,43,44], our result show that prolonged heat treatment (Fig 5 D–5 F) and brief heat exposures (Fig 7 A–7 D) greatly reduces germination and tube development of tobacco pollen (Fig 6). Nevertheless, the degree of high temperature induced inhibition on pollen growth is alleviated in transgenic plant lines. Pollen germination rate and pollen tube growth of transgenic plants are significant higher than those of WT and null transgenic plants under heat-treated conditions (Figs 5–7). The results suggest expressing AtHSP101in tobacco pollen can improve the overall pollen growth in transgenic tobacco lines under heat-stressed conditions.

The observed enhancement in tobacco pollen thermotolerance in AtHSP101 over-expression transgenic plants raised the question whether expressing HSP101 in other species would lead to similar results. Cotton is one of the most important cash crops in US agricultural industry. Reproductive heat sensitivity in cotton has been studied extensively [15–17,19,45–51]. Studies have shown that both cotton pollen development [15,16,49,50] and pollen germination [17,19] are extremely sensitive to high temperatures. Studies have shown that pollen development is ‘a highly controlled sequential process at the proteome level’ [39] and accumulation of HSP proteins during pollen development may play a role in heat stress tolerance [13,39]. To ensure the expression of *AtHSP101* during both pollen development and pollen germination and growth, a constitutive promoter was used in making the *AtHSP101*construct for plant transformation. Western analysis shows moderate amount of AtHSP101 in pollen and leaf tissues of transgenic cotton plants (Fig 4C) while AtHSP101 and HSP101 related component are absence in mature pollen of null lines (Fig 4F).

Consistent with the results of tobacco pollen germination study, heat tolerance of pollen in transgenic cotton lines was greatly enhanced (Figs 8–10). Compared to wild-type and null lines, pollen of AtHSP101 transgenic lines showed great increases in germination rates and pollen tube growth in all three different in vivo thermotolerant assays (Figs 8–10). Our results indicate that expressing AtHSP101in pollen of cotton and tobacco plants increase the overall heat tolerance of mature pollen and hence, may lead to the improvement of overall heat tolerance of reproductive tissues.

Cotton sensitivity to heat stress occurs both to existing and developing reproductive tissues [52]. Boll retention was shown to decrease during a 7-day period of Level 2 stress (canopy temperatures exceeding 30C), and approximately 15 days following the stress exposure [52]. Fisher [15] reported a fertility index of only 30% in a sensitive cultivar exposed to high temperatures the preceding 3 weeks. Other reports of high temperatures (32C+) 15–17 days before anthesis increased sterility of pollen in temperature sensitive male sterile stocks [53,54]. Other studies have showed that sensitivity of pollen germination and pollen tube growth to high temperature are one of the major factors contributing to low boll setting in cotton and low yields in other crops [6,17,33,55]. Reduction in boll size and seed numbers are also common responses of cotton to elevated temperatures [40,48]. The effects of heat stress on cotton production are also observed in our greenhouse studies. The results show significant reductions in boll set in null plants and substantial decrease in the number of seeds per boll in all plants grown under heat-stressed condition (Table 1). These high temperature induced effect on cotton yield components is closely correlated with its negative effects on pollen germination rate and pollen tube growth observed in our in vivo studies (Figs 8–10). The results suggest that enhancing heat tolerance of pollen may lead to improve cotton production under heat stressed environments.

The present study examined the effects of expressing AtHSP101 in cotton on boll production under heat-stresses conditions in both greenhouse and field conditions. Results of greenhouse study have showed an increase in numbers of boll setting and seed productions in transgenic plant lines under heat stressed conditions compared with null plant lines grown under the same conditions (Table 1, Table 2 and Fig 11). In addition, transgenic plants produce larger size of bolls than those of null plant under heat stressed conditions (Table 2). The increase in boll setting in transgenic lines is tightly correlated with the increase in pollen germination and pollen tube growth observed in our in vivo study (Fig 10). The results indicate expressing *AtHSP101* in pollen can enhance heat tolerance of cotton reproductive tissues and reduce cotton yield loss due to high temperature stress.

The effect of the *ATHSP101* transgene on cotton production was examined further in a field heat stressed in Maricopa, Arizona, where the maximum daytime temperatures ranged from 37 to 43°C throughout the boll development and filling period (Fig 13). The results of field study are similar to our greenhouse findings. The AtHSP101 transgenic plants produced more bolls per plant than those null plants (Table 3). Further analysis has shown that the difference in boll production between transgenic and null lines is due to the number of unopened boll set on those plants. There are similar numbers of open bolls and seeds per open boll in the field study. The average 18 seeds per open boll produced in field grown plants in both lines are below the average observed in the control greenhouse studies (25–26 seeds per open boll), but well above the 8–10 seeds per open boll in the hot greenhouse study (Table 1). In general, from the day of flowering, it takes about 45–55 days for a cotton boll to open [56]. The open bolls collected at the time of harvesting (September 28) were likely most set earlier in the season before August 10. The daily temperature data collected at the experimental field (Fig 13) indicate that these open bolls were set under no or moderate heat stress condition, prior to the start of the hot season (11^th^ August). On the other hand, all the unopened bolls were set under extreme heat stressed conditions. The difference in the number of closed bolls per plant between the AtHSP101 transgenic and null plants reflects the different responses of the reproductive tissues of these plants under extreme high temperatures (Table 3). The observed increase in the number of boll produced in transgenic cotton is likely the result of increase in boll set and boll retention rate. The field results confirmed the findings of our greenhouse studies as expressing AtHSP101 in cotton improve cotton productivity under heat stressed conditions.

We have previously reported that optimum pollen germination and rapid tube elongation occurred between 28 and 31°C under 80% relative humidity [17]. Decreased pollen germination occurred at temperatures above 37°C, and decreased tube elongation occurred at temperatures above 32°C. Additionally, we found that pollen from flowers exposed to direct sunlight, taken at 1400 h from field-grown cotton plants, had reduced viability compared with pollen from flowers inside the canopy [17]. This could be attributed to the fact that, for irrigated cotton plants, flowers exposed to full sunlight can experience internal temperatures as high as 39°C, well above the 28 to 31°C optimum for pollen germination. In this study, daytime air temperatures reached 37 to 44°C for more than one month. During this period, flowers shaded within the canopy would have experienced temperatures deleterious to pollen germination and pollen tube development. Flowers directly exposed to the sunlight would have experienced even higher internal temperatures based upon our earlier findings. Plant mapping of boll positions on cotton plants also show that plant grown under heat-stressed field environments set most of bolls within the canopy, as first and/or second position boll on the low fruit branches. This may help explain the poor boll set in the upper half of the canopy for the control null plants versus the transgenic plants.

The results of this study show that over-expression of AtHSP101 in tobacco or cotton pollen improves pollen germination and pollen tube growth under high temperature, enhances the overall heat tolerance of reproductive tissues and reduced yield losses due to high temperature. Enhancing pollen heat tolerance through overexpressing HSP101 holds promise in the development of crop varieties with improved yield production in stressful environments.

## References

[1] Boyer JS. Plant productivity and environment. Science 1982; 218: 443–448. 17808529 10.1126/science.218.4571.443

[2] Abdul-Baki AA, Stommel JR. Pollen viability and fruit set of tomato genotypes under optimum- and high-temperature regimes. HortScience: a publication of the American Society for Horticultural Science 1995; 30: 115–117.

[3] Bajaj M, Cresti M, Shivanna KR. Effects of high temperature and humidity stresses on tobacco pollen and their progeny. Angiosperm pollen and ovules E Ottaviano [et al, editors] 1992: 349–354.

[4] Dane F, Hunter AG, Chambliss OL. Fruit set, pollen fertility, and combining ability of selected tomato genotypes under high-temperature field conditions. Journal of the American Society for Horticultural Science 1991; 116: 906–910.

[5] Halterlein AJ, Clayberg CD, Teare ID. Influence of high temperature on pollen grain viability and pollen tube growth in the styles of Phaseolus vulgaris L. American Society for Horticultural Science Journal of the American Society for Horticultural Science 1980; 105: 12–14.

[6] Harsant J, Pavlovic L, Chiu G, Sultmanis S, Sage TL. High temperature stress and its effect on pollen development and morphological components of harvest index in the C3 model grass Brachypodium distachyon. J Exp Bot 2013; 64: 2971–2983. 10.1093/jxb/ert142 23771979 PMC3697958

[7] Herrero MP, Johnson RR. High temperature stress and pollen viability of maize. Crop science 1980; 20: 796–800.

[8] Prasad PVV, Boote KJ, Allen LH Jr. Adverse high temperature effects on pollen viability, seed-set, seed yield and harvest index of grain-sorghum Sorghum bicolor (L.) Moench are more severe at elevated carbon dioxide due to higher tissue temperatures. Agricultural and forest meteorology 2006; v. 139, no. 3–4: 237–251.

[9] Rao GU, Jain A, Shivanna KR. Effects of high temperature stress on Brassica pollen: viability, germination and ability to set fruits and seeds. Annals of botany 1992; 69: 193–198.

[10] Sakata T, Takahashi H, Nishiyama I, Higashitani A. Effects of high temperature on the development of pollen mother cells and microspores in barley Hordeum vulgare L. Journal of plant research 2000; 113: 395–402.

[11] Weaver ML, Timm H. Screening tomato for high-temperature tolerance through pollen viability tests. HortScience 1989; 24: 493–495.

[12] Gagliardi D, Breton C, Chaboud A, Vergne P, Dumas C. Expression of heat shock factor and heat shock protein 70 genes during maize pollen development. Plant molecular biology 1995; 29: 841–856. 8541509 10.1007/BF00041173

[13] Hopf N, Plesofsky-Vig N, Brambl R. The heat shock response of pollen and other tissues of maize. Plant molecular biology: an international journal on molecular biology, biochemistry and genetic engineering 1992; 19: 623–630. 10.1007/BF00026788 1627775

[14] Volkov RA, Panchuk II, Schoffl F. Small heat shock proteins are differentially regulated during pollen development and following heat stress in tobacco. Plant molecular biology 2005; v. 57, no. 4: 487–502. 15821976 10.1007/s11103-005-0339-y

[15] Fisher WD. Heat induced sterility in upland cotton. Proc Beltwide Cotton Prod Res Conf 1975: 85.

[16] Percy RG, Mayo OL, Ulloa M, Cantrell RG. Registration of AGC85, AGC208, and AGC375 Upland Cotton Germplasm Lines. Crop science 2006; v. 46, no. 4: 1828–1829.

[17] Burke JJ, Velten J, Oliver MJ. In vitro analysis of cotton pollen germination. Agronomy journal 2004; v. 96, no. 2: 359–368. 14723697

[18] Kakani VG, Prasad PVV, Craufurd PQ, Wheeler TR. Response of in vitro pollen germination and pollen tube growth of groundnut (Arachis hypogaea L.) genotypes to temperature. Plant, cell and environment 2002; 25: 1651–1661.

[19] Kakani VG, Reddy KR, Koti S, Wallace TP, Prasad PVV, Reddy VR, et al Differences in in vitro pollen germination and pollen tube growth of cotton cultivars in response to high temperature. Annals of botany 2005; v. 96, no. 1: 59–67. 15851397 10.1093/aob/mci149 PMC4246808

[20] Gurley WB. HSP101: a key component for the acquisition of thermotolerance in plants. Plant cell 2000; 12: 457–460. 10760235 10.1105/tpc.12.4.457 PMC526003

[21] Cooper P, Ho TD, Hauptmann RM. Tissue specificity of the heat-shock response in maize. Plant physiology 1984; 75: 431–441. 16663639 10.1104/pp.75.2.431 PMC1066925

[22] Dupuis I, Dumas C. Influence of temperature stress on in vitro fertilization and heat shock protein synthesis in maize (Zea mays L.) reproductive tissues. Plant physiology 1990; 94: 665–670. 16667763 10.1104/pp.94.2.665 PMC1077283

[23] Frova C, Sari-Gorla M. Quantitative trait loci (QTLs) for pollen thermotolerance detected in maize. Molecular & general genetics: MGG 1994; 245: 424–430. 7808391 10.1007/BF00302254

[24] Ni M, Cui D, Einstein J, Narasimhulu S, Vergara CE, Gelvin SB. Strength and tissue specificity of chimeric promoters derived from the octopine and mannopine synthase genes. Plant journal: for cell and molecular biology 1995; 7: 661–676.

[25] Walker-Peach C, Velten J (1984) Agrobacterium-mediated gene transfer to plant cells: cointegrate and binary vector systems.; Gelvin SB, Schilperoort RA, Verma DPS, editors.

[26] Bayley C, Trolinder N, Ray C, Morgan M, Quesenberry JE, Ow DW. Engineering 2,4-D resistance into cotton. Theoretical and applied genetics 1992; 83 (5): 645–649. 10.1007/BF00226910 24202683

[27] Horsch RB, Fry JE, Hoffmann NL, Eicholtz D, Rogers SG, Fraley RT. A Simple and General Method for Transferring Genes into Plants. Science of the total environment 1985; 227: 1229–1231. 10.1126/science.227.4691.1229 17757866

[28] Otten L, Schilperoort R. A rapid microscale method for the detection of lysipine and nopaline dehydrogenase activities. Biochim Biophys Acta 1978; 527: 497–500. 31918 10.1016/0005-2744(78)90363-7

[29] Burke JJ, O'Mahony PJ, Velten JP, Oliver MJ. Selection procedure for identifying transgenic cells, embryos, and plants without the use of antibiotics. United States Department of Agriculture patents 2005; no. US 6,939,676 B2.

[30] Xin Z, Velten JP, Oliver MJ, Burke JJ. High-throughput DNA extraction method suitable for PCR. Biotechniques 2003; 34: 820–824, 826. 12703307 10.2144/03344rr04

[31] Hong SW, Vierling E. Mutants of Arabidopsis thaliana defective in the acquisition of tolerance to high temperature stress. Proceedings of the National Academy of Sciences of the United States of America 2000; 97: 4392–4397. 10760305 10.1073/pnas.97.8.4392 PMC18252

[32] Queitscha C, Hong SW, Vierling E, Lindquist S. Heat Shock Protein 101 Plays a Crucial Role in Thermotolerance in Arabidopsis. The Plant Cell 2000; 12: 479–492. 10760238 10.1105/tpc.12.4.479 PMC139847

[33] Bokszczanin KL, Fragkostefanakis S. Perspectives on deciphering mechanisms underlying plant heat stress response and thermotolerance. Front Plant Sci 2013; 4: 315 10.3389/fpls.2013.00315 23986766 PMC3750488

[34] De Storme N, Geelen D. The impact of environmental stress on male reproductive development in plants: biological processes and molecular mechanisms. Plant Cell Environ 2014; 37: 1–18. 10.1111/pce.12142 23731015 PMC4280902

[35] Mitchell JC, Petolino JF. Heat stress effects on isolated reproductive organs of maize. J Plant Physiol 1988; 133: 625–628.

[36] Schoper JB, Lambert RJ, Vasilas BL. Pollen viability, pollen shedding, and combining ability for tassel heat tolerance in maize. Crop science 1987; 27: 27–31.

[37] Magnard JL, Vergne P, Dumas C. Complexity and genetic variability of heat-shock protein expression in isolated maize microspores. Plant physiology (Lancaster, Pa) Plant physiology 1996; 111: 1085–1096. 10.1104/pp.111.4.1085 PMC160984 12226349

[38] Young TE, Ling J, Geisler-Lee CJ, Tanguay RL, Caldwell C, Gallie DR. Developmental and thermal regulation of the maize heat shock protein, HSP101. Plant physiology (Lancaster, Pa) Plant physiology 2001; 127: 777–790. 11706162 PMC129251

[39] Chaturvedi P, Ischebeck T, Egelhofer V, Lichtscheidl I, Weckwerth W. Cell-specific analysis of the tomato pollen proteome from pollen mother cell to mature pollen provides evidence for developmental priming. J Proteome Res 2013; 12: 4892–4903. 10.1021/pr400197p 23731163

[40] Boston RS, Viitanen PV, Vierling E. Molecular chaperones and protein folding in plants. Plant Mol Biol 1996; 32: 191–222. 8980480 10.1007/BF00039383

[41] Lee U, Wie C, Escobar M, Williams B, Hong SW, Vierling E. Genetic analysis reveals domain interactions of Arabidopsis Hsp100/ClpB and cooperation with the small heat shock protein chaperone system. Plant Cell 2005; 17: 559–571. 15659638 10.1105/tpc.104.027540 PMC548826

[42] Queitsch C, Hong SW, Vierling E, Lindquist S. Heat shock protein 101 plays a crucial role in thermotolerance in Arabidopsis. Plant Cell 2000; 12: 479–492. 10760238 10.1105/tpc.12.4.479 PMC139847

[43] Kandasamy MK, Kristen U. Ultrastructural responses of tobacco pollen tubes to heat shock. Protoplasma 1989; 153 (1/2): 104–110.

[44] Shivanna KR, Linskens HF, Cresti M. Responses of tobacco pollen to high humidity and heat stress: viability and germinability in vitro and in vivo. Sexual Plant Reproduction 1991; 4: 104–109.

[45] Goswami CL, Dayal J. Evaluation of American cotton varieties on the basis of extent, periodicity and intensity of flower bud and boll abscission. Cotton development 1980; 9: 15–18.

[46] Guinn G. Boll abscission in cotton. Crop physiology: advancing frontiers editor US Gupta 1984: 177–225.

[47] Pettigrew WT. The Effect of Higher Temperatures on Cotton Lint Yield Production and Fiber Quality. Crop science 2008; v. 48, no. 1: 278–285.

[48] Reddy KR, Hodges HF, McKinion JM. Carbon dioxide and temperature effects on pima cotton growth. Agriculture, ecosystems & environment 1995; 54: 17–29.

[49] Reddy KR, Hodges HF, McKinion JM, Wall GW. Temperature effects on Pima cotton growth and development. Agronomy journal 1992; 84: 237–243.

[50] Reddy KR, Hodges HF, Reddy VR. Temperature effects on cotton fruit retention. Agronomy journal 1992; 84: 26–30.

[51] Rodriguez-Garay B, Barrow JR. Pollen selection for heat tolerance in cotton. Crop science 1988; 28: 857–858.

[52] Brown PW, Zeiher CA. Cotton Heat Stress. In: Cotton: A College of Agriculture Report 1997; Series P-108. College of Agriculture, Univ. of Arizona, Tucson, AZ.: 91–104.

[53] Sarvella P. Environmental influences on sterility in cytoplasmic male-sterile cottons. Crop Science 1966; 6: 361–364.

[54] Meyer VG. Some effects of genes, cytoplasm, and environment on male sterility of cotton (Gossypium). Crop Science 1969; 9:237–242.

[55] Snider JL, Oosterhuis DM, Skulman BW, Kawakami EM. Heat stress-induced limitations to reproductive success in Gossypium hirsutum. Physiol Plant 2009; 137: 125–138. 10.1111/j.1399-3054.2009.01266.x 19656331

[56] Reddy KR, Davidonis GH, Johnson AS, Vinyard BT. Temperature regime and carbon dioxide enrichment alter cotton boll development and fiber properties. Agronomy Journal 1999; 91: 851–858. 10541244

[57] Velten J, Schell J. Selection-expression vectors for use in genetic transformation of higher plants. Nucleic Acid Research 1985; 13: 6981–6998. 4059052 10.1093/nar/13.19.6981 PMC322017

[58] Fraley RT, Rogers SG, Horsch RB. Genetic transformation in higher plants. Critical reviews in plant sciences 1986; 4: 1–46.

[59] Koncz C, De Greve H, Andre D, Deboeck F, Van Montagu M, J. S. The opine synthase genes carried by Ti plasmids contain all signals necessary for expression in plants. EMBO Journal 1983; 2: 1597–1603. 11892818 10.1002/j.1460-2075.1983.tb01630.x PMC555329

[60] Bouchez D, Tokuhisa JG, Llewellyn DJ, Dennis ES, Ellis JG. The ocs-element is a component of the promoters of several T-DNA and plant viral genes. EMBO Journal 1989 8: 4197–4204. 2591372 10.1002/j.1460-2075.1989.tb08605.x PMC401614

[61] Velten J, Velten L, Hain R, Schell J. Isolation of a dual plant promoter fragment from the Ti plasmid of Agrobacterium tumefaciens. EMBO Journal 1984; 3: 2723–2730. 16453574 10.1002/j.1460-2075.1984.tb02202.x PMC557759

[62] Bandyopadhyay RS, Bruce WB, Gurley WB. Regulatory elements within the agropine synthase promoter of T-DNA. J Biol Chem 1989; 264: 19399–19406. 2808432

